# Analysis of the Predictors of Mortality from Ischemic Heart Diseases in the Southern Region of Brazil: A Geographic Machine-Learning-Based Study

**DOI:** 10.5334/gh.1371

**Published:** 2024-11-27

**Authors:** Amanda de Carvalho Dutra, Lincoln Luis Silva, Isadora Martins Borba, Amanda Gubert Alves dos Santos, Diogo Pinetti Marquezoni, Matheus Henrique Arruda Beltrame, Rogério do Lago Franco, Ualid Saleh Hatoum, Juliana Harumi Miyoshi, Gustavo Cezar Wagner Leandro, Marcos Rogério Bitencourt, Oscar Kenji Nihei, João Ricardo Nickenig Vissoci, Luciano de Andrade

**Affiliations:** 1 Graduation Program in Health Sciences, State University of Maringa, Parana, Brazil; 2 Department of Medicine, State University of Maringa, Parana, Brazil; 3 Department of Clinical Analysis and Biomedicine, State University of Maringa, Parana, Brazil; 4 São Paulo State University, São Paulo, Brazil; 5 Graduation Program in pharmaceutical sciences, State University of Maringa, Parana, Brazil; 6 Education, Letters and Health Center, State University of the West of Paraná, Parana, Brazil; 7 Duke Global Health Institute, Duke University, Durham, North Carolina, United States

**Keywords:** Myocardial Ischemia, Spatial Analysis, Supervised Machine Learning, Epidemiology

## Abstract

**Background:**

Mortality due to ischemic heart disease (IHD) is heterogeneously distributed globally, and identifying the sites most affected by it is essential in developing strategies to mitigate the impact of the disease, despite the complexity resulting from the great diversity of variables involved.

**Objective:**

To analyze the predictability of IHD mortality using machine learning (ML) techniques in combination with geospatial analysis in southern Brazil.

**Methods:**

Ecological study using secondary and retrospective data on mortality due to ischemic heart disease (IHD) obtained from the Mortality Information Systems (SIM-DATASUS) de 2018 a 2022, covering 1,191 municipalities in the states of Paraná (399), Santa Catarina (295), and Rio Grande do Sul (497). Ordinary Least Squares Regression (OLS), Geographically Weighted Regression (GWR), Random Forest (RF), and Geographically Weighted Random Forest (GWRF) analyses were performed to verify the model with the best performance capable of identifying the most affected sites by the disease based on a set of predictors composed by variables of procedures and access to health.

**Results:**

In the analyzed period, there were 59,093 deaths, 65% of which were men, 82.7% were white, and 72.8% occurred between 60 and 70 years of age. Ischemic heart disease presented the highest mortality rates in the northwest and north regions of the state of Paraná, and in the central-east, southwest and southeast regions of Rio Grande do Sul, the latter state accounting for 41% of total deaths. The GWRF presented the best performance with R^2^ = 0.983 and AICc = 2298.4, RMSE: 3.494 and the most important variables of the model in descending order were electrocardiograph rate, cardiac catheterization rate, access index to hemodynamics, access index of pre-hospital mobile units, cardiologists rate, myocardial scintigraphy rate, stress test rate, and stress echocardiogram rate.

**Conclusion:**

The GWRF identified spatial heterogeneity in the variation of geographic predictors, contrasting the limitation of linear regression models. The findings showed patterns of vulnerability in southern Brazil, suggesting the formulation of health policies to improve access to diagnostic and therapeutic resources, with the potential to reduce IHD mortality.

## Introduction

Ischemic heart disease (IHD) is the most prevalent form of cardiovascular disease and the leading cause of death globally, with a 39.4% increase in 2022 (1,2,3). This condition stands out in public health discussions in countries such as Russia, the United States, Ukraine, Germany, and Brazil, where it represents more than half of the proportional mortality (1,2,3,4,5). Ischemic heart disease mortality is multifactorial, involving lifestyle changes, population aging, and lack of access to health services, representing a challenge for reducing morbidity and mortality, especially in Global South countries (6).

Survival of an acute IHD event depends on immediate and specialized interventions, the effectiveness of which varies depending on resource availability and patient location (7,8,9,10). Public health studies indicate the relationship between access to health services, socioeconomic disparities, and incidence of heart disease (7,8,9,10,11).

Previous studies have shown that machine learning (ML) has high performance in predicting cardiovascular disease at the individual level (12,13,14). However, despite the significant advance in the application of ML to predict cardiovascular diseases at the individual level, there is a relevant gap in the ecological prediction of IHD mortality, that is, in the analysis of data aggregated by geographic regions that consider variables such as coverage and access to health. Ecological prediction becomes fundamental because it allows the formulation of more effective and targeted public policies, especially in vulnerable areas where the incidence of IHD is high and resources are scarce (15,16).

The spatial analysis of these phenomena reveals important information for reducing IHD mortality, highlighting the influence of the distribution of health resources and the need for equity in access to medical care, especially in vulnerable areas (17). The use of geographic information systems (GIS) allows for assessing accessibility to health services, providing insight into the distribution and effectiveness of actions and resources (7,8,9,10,11,18).

The application of machine learning techniques, for spatial prediction of deaths from IHD, complements spatial analysis by allowing detailed analyses of spatiotemporal dynamics and the effects of contextual variables on clinical outcomes, with greater sensitivity and specificity than human capabilities (19,20,21,22).

Therefore, this research aims to explore the prediction of IHD mortality using machine learning (ML) techniques in combination with geospatial analysis in southern Brazil, aiming to improve the understanding of the factors that lead to delayed care and consequently influence high IHD mortality in different locations.

## Methodology

### Study design and site

This is a cross-sectional, descriptive, and ecological study, based on geospatial analysis and ML tools, using secondary IHD mortality data in southern Brazil between 2018 and 2022, and possible associated factors. The methodological quality was guaranteed following the *Transparent Reporting of a multivariable prediction model for Individual Prognosis or Diagnosis* (TRIPOD) recommendations (23).

The southern region of Brazil had an estimated population of 29,933,315 inhabitants in 2022, corresponding to about 30% of the national population, distributed over a total area of 576,774 km^2^ divided between 1,191 municipalities in the states of Paraná (399), Santa Catarina (295), and Rio Grande do Sul (497) (24). The region is situated between the latitudes of 22°30’58” and 26°43’00”, and longitudes 48°05’37”, and 54°37’08” (25).

### Data source

#### Outcome variable

Two outcome variables were used in this study. The variable mortality rate due to IHD was calculated by the average number of deaths due to IHD divided by the population aged 30 to 70 years and multiplied by 100,000 between 2018 and 2022. This variable was used in the analysis of Ordinary Least Square Regression (OLS) and Geographically Weighted Regression (GWR). The choice of this age group follows the guidelines of the American Heart Association, which indicates a higher probability of death in this age group (26). The number of deaths from IHD in individuals aged 30 to 70 years in the municipalities of the southern region of Brazil from 2018 to 2022 was obtained from the Mortality Information System (SIM) (27), considering deaths whose underlying cause was classified under codes I20 to I25 of the International Statistical Classification of Diseases and Related Health Problems (ICD) (10).

For the Random Forest (RF) and Geographically Weighted Random Forest (GWRF) analyses, the outcome variable was divided into three datasets. The mortality rates of the year 2020 were used for training, those of 2021 for validation, and those of 2022 for testing.

#### Independent variables

Independent variables related to health accessibility and procedures are described in Table 1. The variables were created using rates to normalize the data, allowing for a better understanding of the proportions of the procedures. The variable rate of cardiologists was created by dividing the number of cardiologists per 1,000 inhabitants. The number of electrocardiographs were calculated by dividing the number of electrocardiographs per 10,000 inhabitants. Similarly, the procedures of cardiac catheterization, echocardiography, exercise stress test, and myocardial scintigraphy were calculated by dividing the number of procedures performed per 10,000 inhabitants (34).

**Table 1:** Data source.


VARIABLES	PERIOD	CONSTANT	SOURCE

Mortality from Ischemic Heart Diseases (IHD)	2018–2022	100.000	27

Population	2018–2022	100.000	32

Electrocardiograph rate	2018–2022	10.000	33

Cardiac catheterization rate	2018–2022	10.000	34

Access index to hemodynamics	2018–2022	E2SFCA	33

Access index of pre-hospital mobile units	2018–2022	E2SFCA	33

Cardiologists rate	2018–2022	1.000	33

Myocardial scintigraphy rate	2018–2022	10.000	34

Stress test rate	2018–2022	10.000	34

Stress echocardiogram rate	2018–2022	10.000	34

Shapefile from southern Brazil	2022	1191	35


The variables accessibility to hemodynamics and accessibility to pre-hospital mobile units were created using the Enhanced Two-Step Floating Catchment Area (E2SFCA) method. This method calculates the ratio of available health services to the population in a given area and adjusts that ratio by geographic distance. The E2SFCA results in an index that incorporates the decreasing influence of services as distance or travel time increases, using a decay function that adjusts the influence about distance (buffer with distance of 60 km based on the transport network) and travel time (60 minutes) (28,29). Health services closer to the city center have a greater weight in total accessibility, with the time chosen based on the guidelines for the treatment of acute ST-elevation myocardial infarction (26,30,31).

### Data analysis

#### Spatial dependency analysis

Initially, data from 1,191 municipalities in the southern region of Brazil were linked to the cartographic base made available by IBGE (35). Next, an empirical Bayesian estimator based on the first-order *queen* neighborhood matrix in the GeoDA software (version 1.12.0) was used to create the smoothed IHD mortality rates in order to minimize possible spatial discrepancies (36).

Moran’s I Index was applied to analyze global spatial autocorrelation to verify the existence of spatial dependence for the distribution of mortality. Values less than zero indicated negative correlation, equal to zero indicated no correlation, and greater than zero indicated positive correlation (37). One of the main limitations of the Global Moran Index is the inability to identify groups of municipalities with high or low mortality. However, to solve this limitation, the Local Indicators of Spatial Association (LISA) technique was applied, capable of demonstrating the existence of local spatial *clusters* with high or low mortality rates, identifying the regions that most contribute to the existence of spatial autocorrelation (37,38).

The clusters identified by LISA were categorized as high-high (HH), that is, municipalities with high mortality from IHD and with neighbors also with high mortality rates. The low-low type (LL) refers to the inverse, that is, low mortality surrounded by low mortality (7).

Next, Local Bivariate Moran’s analyses were performed to verify whether the independent variables had spatial dependence when analyzed in pairs with the mortality rate (39). This type of analysis is particularly useful for understanding how two different variables interact spatially, revealing patterns of concentration or dispersion that may not be evident when considering each variable separately.

These analyses were performed in the GeoDa software (version 1.22) and considered statistically significant when p < 0.05. The results were transferred to the Qgis software (version 3.14) for the elaboration of chloroplastic maps.

#### Global modeling algorithms without spatial components

The following global models do not use the spatial component and serve as elements of comparison with models that use spatial components to verify the influence of spatiality on the distribution of mortality. The global models initially used were Ordinary Least Squares (OLS) and Random Forest (RF). Ordinary Least Squares is a method that estimates the angular coefficients of each independent variables in relation to the dependent variable of a linear model (40). This analysis depends on restrictive assumptions about linearity, homoscedasticity, and normality of residuals, which may impair their performance in nonlinear relationships or heterogeneous variance of errors (9,39,40). In addition, it does not directly assess the importance of independent variables, limiting their usefulness in certain analytical contexts.

Random Forest is a non-parametric global machine learning technique, which constructs a set of decision trees for classification or regression (41). In addition, RF stands out for its ability to assess the relative importance of each independent variable without relying on standardized coefficients, being effective in modeling complex and nonlinear relationships between variables (40,41,42).

To examine local collinearity in a regression model, we also calculated local Variance Inflation Factors (VIF’s) for each independent variable. Local collinearity problems in the regression model are generally identified when VIF’s greater than five are found at any location for any independent variables (43).

#### Local modeling algorithms with spatial components

For analyses with spatial dependence, Geographically Weighted Regression (GWR) and Geographically Weighted Random Forest (GWRF) were used.

Geographically Weighted Regression is a technique that estimates spatially variable regression coefficients, using weighted OLS based on the distance between each observation and the location being modeled, thus capturing spatial heterogeneity in the data (44). The matrix of spatial weights is crucial in GWR for the correct estimation of regression parameters. The spatial weight function was implemented using the Gaussian method, which establishes the relationship between weight and distance through a monotone decreasing function (44). Bandwidth size controls the degree of attenuation of weights with increasing distance (45).

Geographically Weighted Random Forest is a machine learning algorithm that combines GWR’s ability to account for spatial dependence of variables with RF’s robustness in handling high-dimensional data and capturing complex nonlinear relationships (46). The algorithm segments the study area into several smaller regions, modeling each separately with RF. Then, it estimates the spatial variation between dependent and independent variables in each region. Geographically Weighted Random Forest uses the same GWR calculation to determine bandwidth and kernel selection (47,48).

To implement the global (OLS and RF) and local (GWR and GWRF) models, the ‘h2o’, ‘GWmodel’ and ‘SpatialML’ packages of the R programming language were used (49). To evaluate the fit of the models, the highest value of the coefficient of determination (R^2^) and the lowest values of *Root Mean Squared Error* (RMSE) and *Mean Absolute Error* (Mae) were used as evaluation metrics.

#### Test, training and validation data

For the analyses using RF and GWRF, the data were divided into three distinct sets, corresponding to complete years, maintaining the same variables in all sets. Specifically, the split was performed as follows: the training set used data from the year 2020, the validation set used data from the year 2021, and the test set used data from the year 2022.

For RF, the 10-part cross validation method was used to determine the best hyperparameter combinations of 200 models. For the GWRF, the RF model with the lowest Root Mean Squared Error (RMSE) in the validation dataset was selected.

For the interpretation of RF and GWRF model prediction, we implemented several explainable machine learning strategies (50). Initially, partial dependence plots were employed to investigate the direction and nature of the relationships, taking into account the average effects of the independent variables (51,52,53). Subsequently, the significance of each covariate was determined through the global permutation of its values, wherein the decreased model performance indicates the relevance of the covariate (51). Additionally, the importance of the independent variables were measured at the local level, similar to the traditional RF permutation methodology, classifying the variables by the increase in mean square error (IncMSE) (51,54,55) enabling the analysis of the geographic impact of each variable for each locality. Higher IncMSE values correspond to the greater importance of the predictor variable for the municipality in question.

Finally, the OLS, RF, GWR and GWRF models were compared and classified with the best performance based on the highest value of R^2^, and lowest values of corrected Akaike Information Criterion (AICc) and Moran Residues.

## Results

Between 2018 and 2022, there were 59,093 deaths attributed to IHD among individuals aged 30 to 70 years in southern Brazil. The mortality distribution in the states of Paraná (n = 21,565, 36.5%), Santa Catarina (n = 13,296, 22.5%), and Rio Grande do Sul (n = 24,237, 41.0%) in the analyzed period revealed a higher number of deaths among men (n = 38,864, 65.7%), Caucasian (n = 48,914, 82.7%), and aged between 60 and 70 years (n = 43,081, 72.8%), as shown in Table 2.

**Table 2:** Number of deaths attributed to IHD in subjects aged 30 to 70 years in the Southern region of Brazil, 2018–2022.


VARIABLES	SOUTH REGION N = 59,093			

	PARANÁ N = 21,565 (36.5%)	SANTA CATARINA N = 13,296 (22.5%)	RIO GRANDE DO SUL 24,232 (41.0%)	TOTAL N = 59,093 (100%)

**Gender**				

Male	14,171 (65.7%)	9,034 (68.0%)	15,657 (64.6%)	38,859 (65.8%)

Female	7,394 (34.3%)	4,262 (32.0%)	8,575 (35.4%)	20,230 (34.2%)

**Race**				

White	16,299 (75.6%)	11,894 (89.5%)	20,721 (85.5%)	48,912 (82.8%)

Non-white	5,266 (24.4%)	1,402 (10.5%)	3,511 (14.5%)	10,181 (17.2%)

**Age group (years)**				

30–40	448 (2.1%)	241 (1.8%)	362 (1.5%)	1,051 (1.8%)

40–50	1,590 (7.4%)	970 (7.3%)	1,403 (5.8%)	3,962 (6.7%)

50–60	4,137 (19.2%)	2,761 (20.8%)	4,105 (16.9%)	10,999 (18.6%)

60–70	15,390 (71.4%)	9,324 (70.1%)	18,362 (75.8%)	43,081 (72.9%)


**Figure 1:**
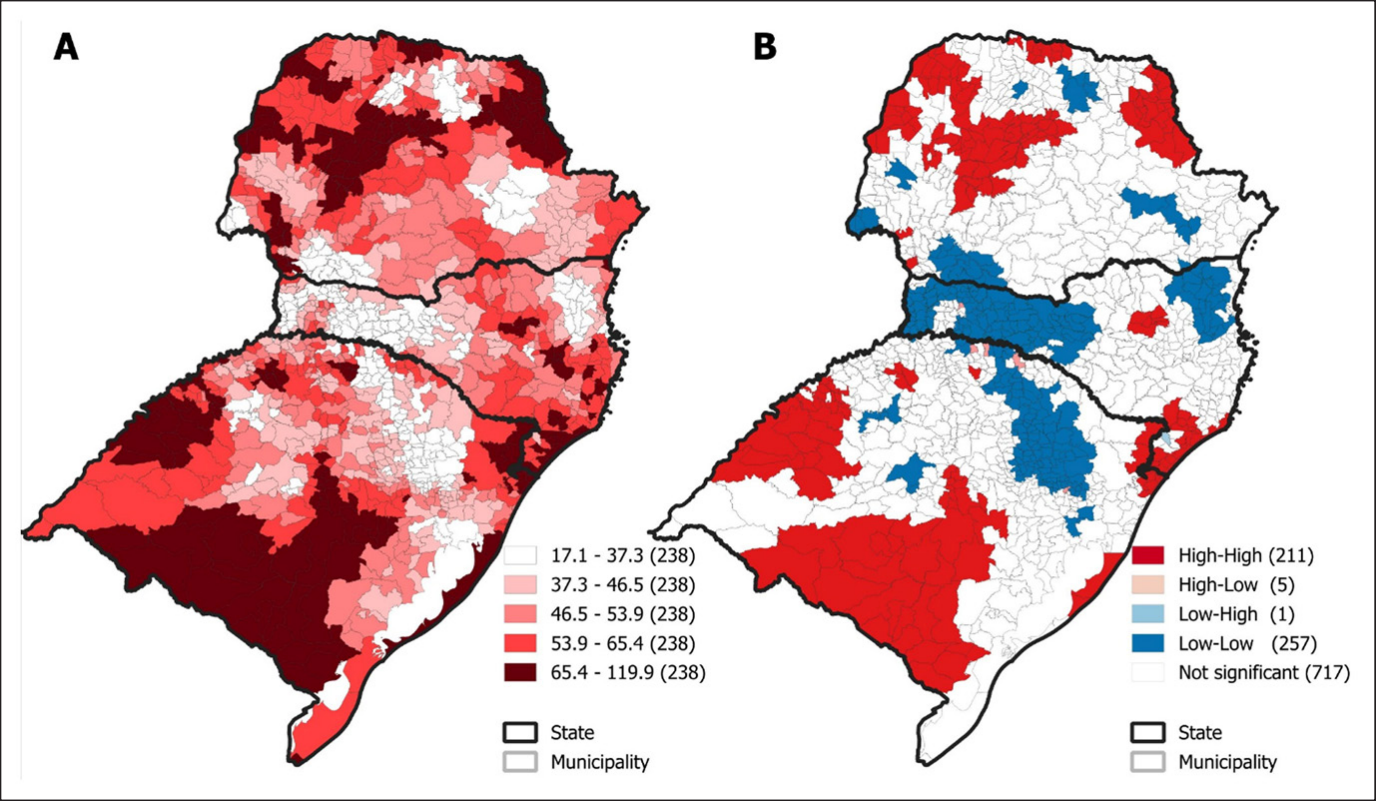
Spatial distribution of IHD mortality rates in the 1,191 municipalities of southern Brazil, and their clusters. **1A.** IHD mortality rates per adjusted population/100,000 inhabitants in Southern Brazil, 2018 to 2022; **1B.** Analysis of Local Indicators of Spatial Association (LISA) indicating clusters according to distribution patterns of high and low IHD mortality rates.

Figure 1A shows the average percentages of IHD mortality between 2018 and 2022. It is observed that all municipalities presented mortality rates ranging between 17.1 and 119.9 per 100,000 inhabitants. The municipalities with high rates were mainly in the states of Paraná and Rio Grande do Sul. The annual average was 51.82 (SD: 16.84) per 100,000 inhabitants in the period.

The univariate global Moran’s analysis indicated a positive spatial autocorrelation (Moran’s I = 0.750; p < 0.01), indicating the existence of spatial dependence in the distribution of deaths from IHD in the southern region. In addition, the LISA analysis detected 211 (17.7%) sets of municipalities with high-high standard (HH), that is, municipalities with high IHD mortality rates are surrounded by neighboring municipalities with high mortality rates (Figure 1B). On the other hand, municipalities of the low-low type (LL) are those that have low mortality rates also surrounded by municipalities with low rates.

In the bivariate analysis of global Moran’s, a significant positive spatial correlation was identified between the mortality rate from IHD and the variables *cardiac catheterization rate* (Moran I = 0.09, p < 0.001), *access index of pre-hospital mobile units* (Moran I = 0.05, p < 0.001), and *stress test rate* (Moran I = 0.10, p < 0.001). On the other hand, a negative correlation was observed between the IHD mortality rate and the variables *electrocardiograph rate* (Moran I = –0.15, p < 0.001), *access index to hemodynamics* (Moran I = –0.08, p > 0.001), *cardiologists rate* (Moran I = –0.08, p < 0.001), and *stress echocardiogram rate* (Moran I = –0.05, p < 0.001).

**Figure 2:**
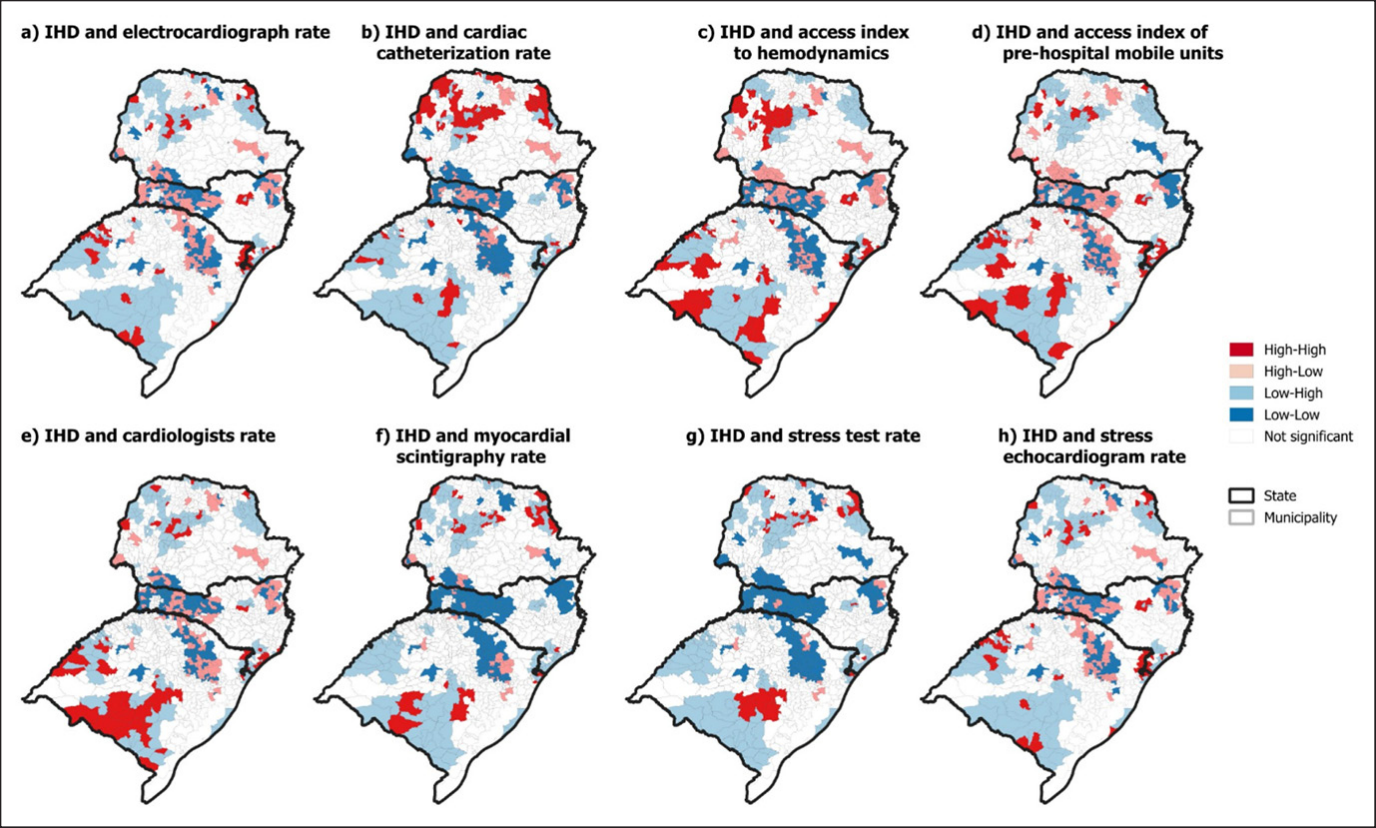
Bivariate Local Moran of ischemic heart disease (IHD) in the south region of Brazil.

As shown in Figure 2, the sets of municipalities are presented when analyzed by the Bivariate Local Moran. The sets of HH-type municipalities are those with high mortality rates surrounded by municipalities with high rates of a given variable. All variables presented this pattern; however, it is observed in greater quantity with the variables *IHD* × *cardiac catheterization rate* (105), *IHD* × *access index to hemodynamics* (86), *IHD* × *access index of pre-hospital mobile units* (70), *IHD* × *electrocardiograph rate* (57), *IHD* × *cardiologists rate* (46)*, IHD* × *myocardial scintigraphy rate* (33), *IHD* × *stress rate test* (32), and *stress echocardiogram rate* (13). The HH clusters formed by IHD mortality rates and cardiac catheterization rate, access index to hemodynamics, and access index of pre-hospital mobile units are concentrated in the northwest and north regions of Paraná, and central-east, southwest and southeast regions of Rio Grande do Sul.

Table 3 shows the results of OLS, GWR, RF, and GWRF regressions. The GWRF model showed the best performance in terms of R^2^ (0.983), AICc (2298.4), RMSE: 3.494, and Moran residues (–0.151).

**Table 3:** Results of global and local multivariate analysis of IHD mortality rates of municipalities in southern Brazil.


OLS					GWR					RF				GWRF			
			
VARIABLE	COEFFICIENT	Std. ERROR	T-VALUE	PROBABILITY (>|t|)	MIN	1 st QU	MEDIAN	3RD QU	MAX	MIN	MAX	MEAN	Std	MIN	MAX	MEAN	Std

**Electrocardiograph rate**	–1.062	0.217	–4.879	0.000***	–2.387	–1.035	–0.407	0.220	2.745	3744.675	99887.77	99959.000	19040.083	0.364	3239.136	270.614	425.543

**Cardiac catheterization rate**	0.229	0.065	3.500	0.000***	–1.441	0.021	0.203	0.410	1.232	4755.092	30693.36	14577.569	5217.347	0.246	7015.351	319.890	598.804

**Access index to hemodynamics**	–1.594	0.478	–3.330	0.000***	–23.272	–2.723	–0.959.	0.613	6.248	4150.982	30591.54	12584.163	5614.0434	0.000	4039.209	277.454	460.952

**Access index of pre-hospital mobile units**	–0.090	0.042	–2.148	0.031*	–2.275	–0.320	–0.034	0.107	0.910	3988.512	30506.97	13369.577	5293.7682	0.156	4500.410	307.706	510.182

**Cardiologists rate**	–6.409	2.429	–2.639	0.008**	–35.999	–8.610	–2.799	1.230	16.051	1988.913	9959.448	5098.821	1665.442	0.000	4643.060	140.773	339.669

**Myocardial scintigraphy rate**	0.189	0.462	0.410	0.681	–25.294	–2.034	0.137	1.464	20.759	370.279	11722.93	3081.451	2522.169	0.000	2510.193	56.666	184.920

**Stress test rate**	3.065	1.197	2.560	0.010*	–273.639	–6.438	0.912	5.158	400.816	62.118	6995.904	2214.965	1598.3471	0.000	1426.459	36.062	122.344

**Stress echocardiogram rate**	–1.082	0.893	–1.212	0.225	–50.319	–1.381	0.478	3.015	26.689	683.792	5482.294	2125.966	964.8613	0.000	2581.386	38.119	177.638

**R^2^**	0.066				0.656					0.058				0.983			

**Aicc**	10034.81				9074.02					4550.3				2298.4			

**Moran Residues**	0.694				0.408					0.500				–0.151			


Signif. codes: 0 ‘***’ 0.001 ‘**’ 0.01 ‘*’ 0.05 ‘.’ 0.1.

**Figure 3:**
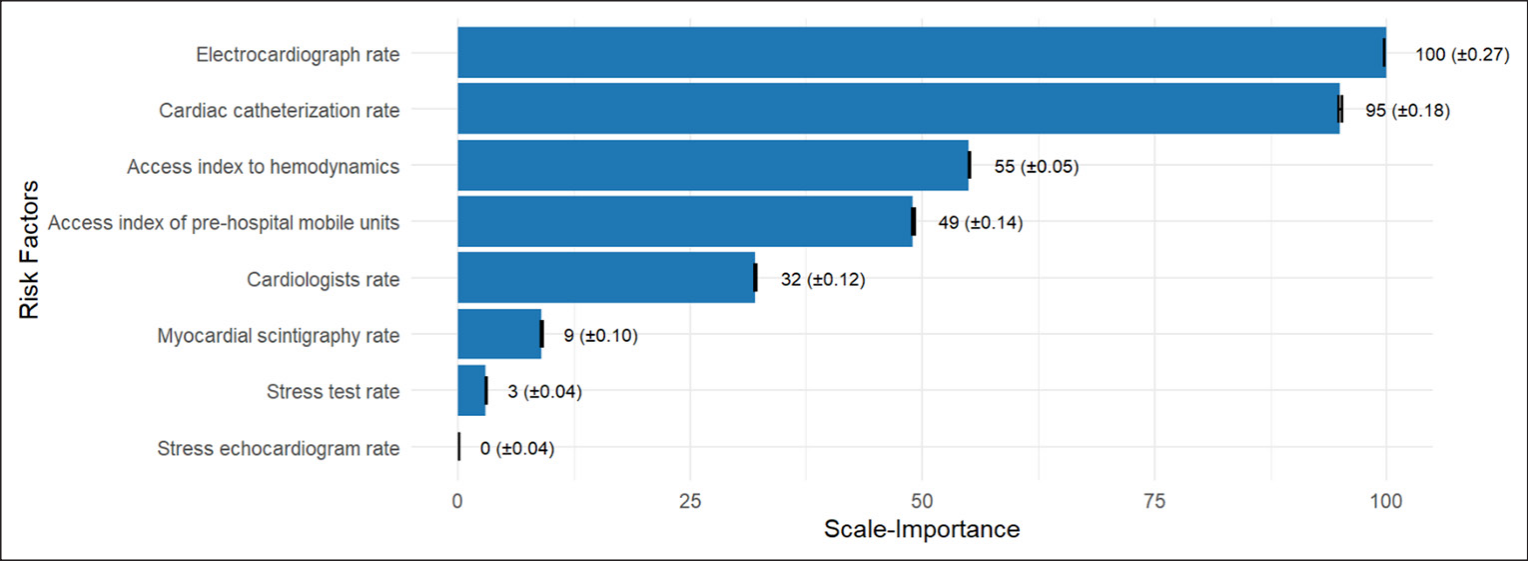
Importance of the variable according to the incMSE.

The GWRF model showed the mean local importance values (%IncMSE) in descending order: electrocardiograph rate, Cardiac catheterization rate, Access index to hemodynamics, Access index of pre-hospital mobile units, Cardiologists rate, Myocardial scintigraphy rate, Stress test rate, and Stress echocardiogram rate (Figure 3).

**Figure 4:**
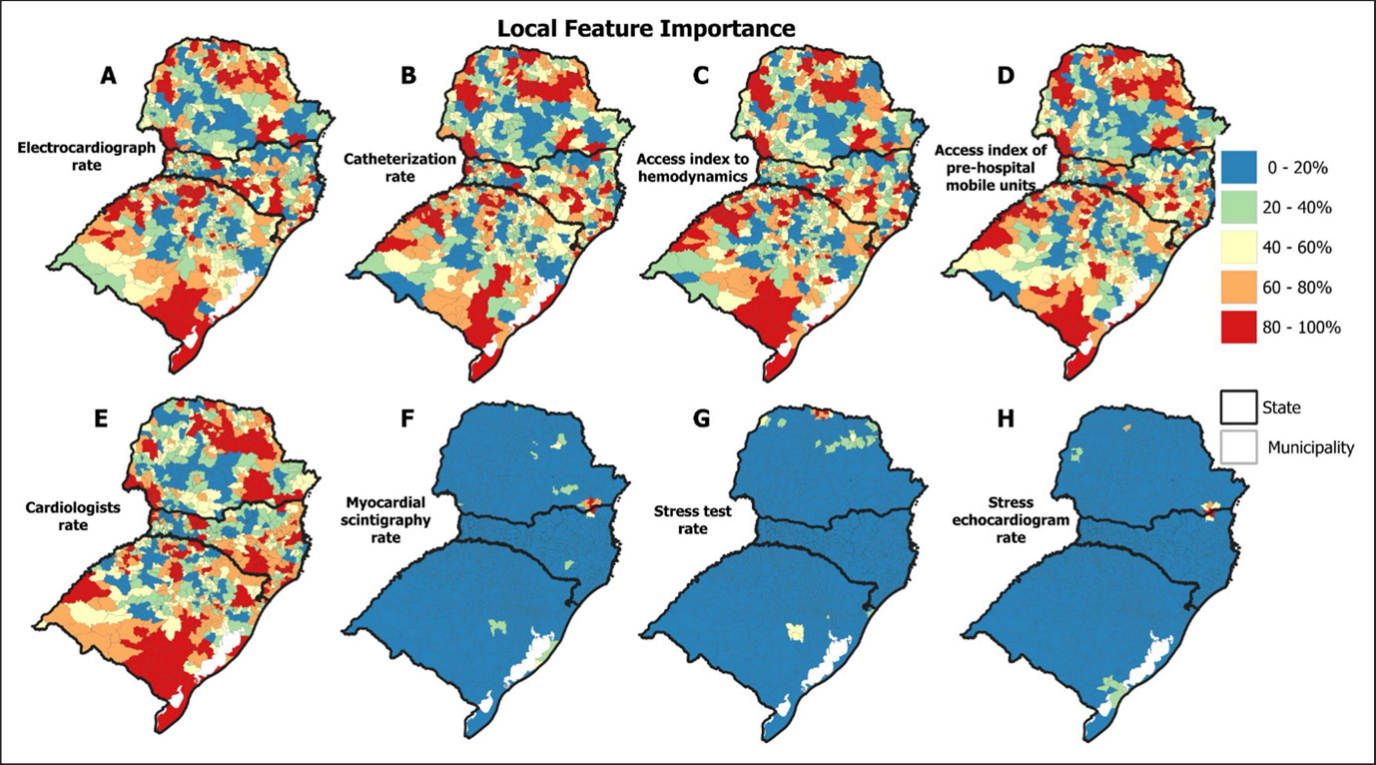
Spatial distribution of predictor variables’ importance in the GWRF model.

Figure 4 shows the importance values of the predictor variables in the GWRF analysis, in which warmer colors indicate greater importance and cooler colors indicate less importance. The variables *stress echocardiogram rate, myocardial scintigraphy rate* and *stress test rate* showed low importance (0–20%) for 1175 (98.65%), 1168 (98.06%) and 1154 (96.89%) municipalities.

On the other hand, the variables *electrocardiograph rate, access index to hemodynamics, cardiac catheterization rate, cardiologists’ rate* and *access index of pre-hospital mobile units* stood out with high importance (80–100%) in 380 (31.90%), 375 (31.48%), 373 (31.31%), 367 (30.81%), and 365 (30.64%) municipalities. These municipalities are distributed in the North, Northwest and Eastern Center, South and Mountain regions of Santa Catarina, and Southeast, Southwest, Northwest and Northeast of Rio Grande do Sul, respectively.

**Figure 5:**
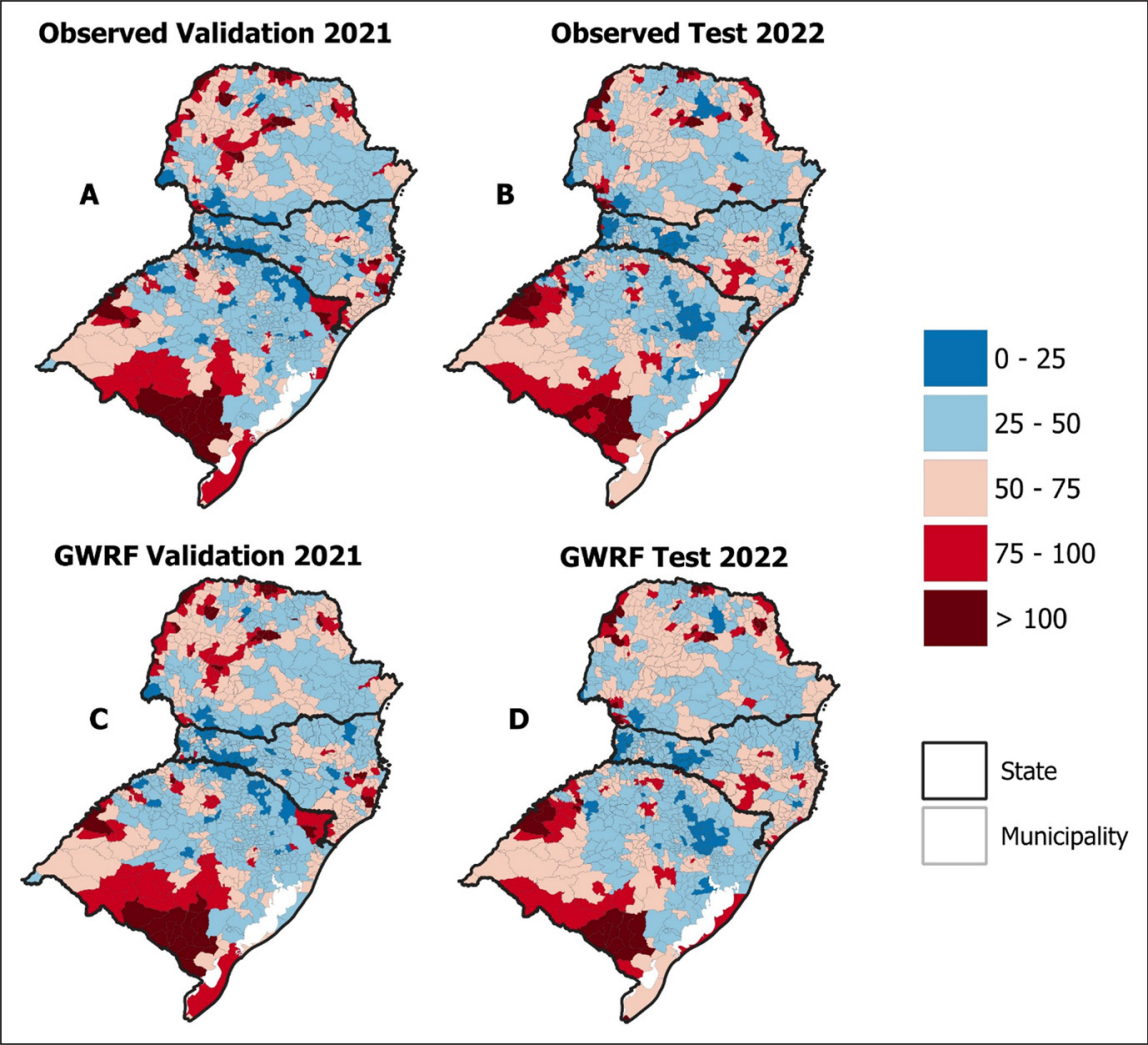
Ischemic heart disease mortality rates predicted using GWRF.

As for the validation of the GWRF, as illustrated in Figure 5, it is observed that the GWRF estimated mortality values close to those observed. ‘Observed Validation 2021’ represents the distribution of mortality observed in 2021, while ‘GWRF Validation 2021’ refers to the use of the trained algorithm to make final adjustments to the model. ‘Observed Test 2022’ represents the distribution of mortality observed in 2022. ‘GWRF Test 2022’ is the application of the refined model to estimate mortality rates for the year 2022. The results show that the mortality rates estimated for 2022 by the model are very similar to those observed, thus validating the predictive capacity of the GWRF.

**Figure 6:**
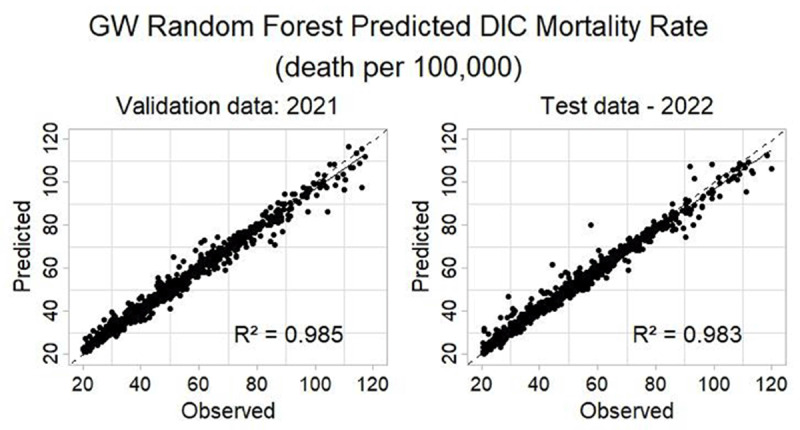
Determination coefficients of validation and test data using Geographically Weighted Random Forest to predict IHD Mortality Rate.

Figure 6 shows the comparison of observed and expected values when analyzed by the GWRF. When analyzing the predicted and observed values, the GWRF presented an R^2^ of 0.985 for the 2021 validation data, and 0.983 for the 2022 test, indicating an almost perfectly linear accuracy.

The results obtained demonstrate the predictive capacity of the GWRF model in estimating the mortality rate by IHD. These indicators reveal that the model is able to explain more than 98% of the variability in the data.

## Discussion

The bulwark of this study lies in its ability to support targeted and evidence-based public health policies. Using advanced machine learning techniques and geo-artificial intelligence, such as GWRF (46), we were able to identify and map predictors associated with higher IHD mortality in municipalities in the southern region of Brazil, which in turn can guide more effective and equitable public health interventions.

Our main findings were the identification of predictors associated with higher IHD mortality, and varying spatially in the southern region of Brazil, confirming that socio-demographically disadvantaged territories have higher IHD mortality rates, in direct relationship with the availability of cardiologists, diagnostic tests and accessibility to hemodynamic centers. Most deaths from IHD occurred in white individuals in the southern region of Brazil, possibly due to the predominantly white demographic composition of the region.

Machine learning has been used with some frequency to analyze mortality from ischemic heart disease and identify the critical risk factors that contribute to higher mortality rates (56,57). By utilizing more advanced and geographically weighted ML techniques, we were able to find out that different factors are associated with IHD mortality rates at different spatial locations. This reveals that the interrelationships between risk factors and IHD mortality are complex and dynamic and cannot be adequately reflected by traditional linear methods (58). However, this approach can be criticized for its complexity and need for detailed spatial data, which is not always available (57).

The results of this study corroborate the current literature that emphasizes the importance of technological advances and early diagnosis in reducing IHD deaths (59,60,61,62,63). These studies show that spatial patterns related to the number of cardiologists, the availability of tests (electrocardiogram, catheterization, myocardial scintigraphy, exercise testing, stress echo), and accessibility to hemodynamics centers and pre-hospital ambulances are directly related to IHD mortality rates (59,61).

The study of Virani et al. (2023) reinforces the importance of the ECG and catheterization in the diagnosis and treatment of IHD, while Rafie (2021) addresses myocardial scintigraphy as fundamental in the evaluation of myocardial perfusion and risk stratification in patients with IHD (63,64). These studies reinforce our findings on the importance of a well-structured and equipped health system with rapid access to diagnostic tests and therapeutic interventions. The epidemiological report highlights the high prevalence of IHD in Brazil and the need for investments in its treatment in the way of infrastructure and human resources, such as accessibility to hemodynamics and the number of cardiologists (65).

The majority of deaths due to IHD (82.8%) occurred in white individuals in the southern region of Brazil, a significantly higher proportion than that observed in non-white individuals (17.2%). This pattern can be partially explained by the predominantly white demographic composition of the region due to its colonial history (66). However, other factors such as access to health services, lifestyle habits and genetic predisposition may also influence the occurrence of IHD in different population groups (67).

Similarly to our findings, previous studies indicate that age and education level are associated with cardiovascular disease comorbidity (59,61). Populations over 60 years of age have high IHD mortality rates, highlighting the importance of mapping age-related spatial clusters to improve predictions and planning of highly complex care and the responsiveness of the health system (59,68). In Spain, Benett et al. (2022) found a higher incidence of cardiovascular diseases in disadvantaged areas (69), highlighting the need for special attention to these regions.

Despite having high socioeconomic indicators, such as human development, per capita income and access to basic services, the Southern region still faces significant social inequalities, especially in rural areas (67). These rural areas are more vulnerable to IHD mortality (16), highlighting that low accessibility to health care is associated with increased mortality. Improving access to care can reduce geographic disparities in AMI mortality (11,70).

This study has some limitations, since this research was based on secondary data and depended on the accuracy of the primary records. However, the quality of secondary data provided by the Brazilian Ministry of Health has improved over time, becoming the best available source for obtaining public health information.

Future studies could benefit from the integration of longitudinal data and the use of machine learning techniques to predict changes over time and apply them in other geographic regions, and for other chronic diseases, expanding the understanding of the spatiotemporal interrelationships between risk factors and health outcomes. This could significantly contribute to the creation of public health policies more focused on improving accessibility to services and investing in prevention, including pre-hospital mobile units, hemodynamic services, cardiologist training, and complementary tests such as scintigraphy, exercise testing, and stress echocardiography, aiming to reduce IHD mortality.

## Conclusions

Geographically weighted random forest was effective in capturing spatial heterogeneity by analyzing the variation in the importance of predictors over geographic space, contrasting with the limitation of linear regression models that offer only local coefficients. Our findings have important political implications by revealing geographic patterns of vulnerability in the southern region of Brazil, enabling the formulation of evidence-based health policies to improve access to diagnostic and therapeutic resources and thus contribute to decreasing IHD mortality.

## Data Accessibility Statement

The data is available in the publicly accessible online repository: https://figshare.com/s/5d66435684b6dc3ab417.

## References

[1] Bonita R, Magnusson R, Bovet P, Zhao D, Malta DC, Geneau R, et al. Country actions to meet UN commitments on non-communicable diseases: A stepwise approach. The Lancet. 2013; 381:575–584. DOI: 10.1016/S0140-6736(12)61993-X 23410607

[2] Roth GA, Johnson C, Abajobir A, Abd-Allah F, Abera SF, Abyu G, et al. Global, regional, and national burden of cardiovascular diseases for 10 causes, 1990 to 2015. Journal of the American College of Cardiology. 2017; 70:1–25. DOI: 10.1016/j.jacc.2017.04.052 28527533 PMC5491406

[3] Nowbar AN, Gitto M, Howard JP, Francis DP, Al-Lamee R. Mortality from ischemic heart disease: Analysis of data from the World Health Organization and Coronary Artery Disease Risk Factors from NCD Risk Factor Collaboration. Circ: Cardiovascular Quality and Outcomes. 2019; 12:e005375. DOI: 10.1161/CIRCOUTCOMES.118.005375 31163980 PMC6613716

[4] Mensah GA, Fuster V, Murray CJL, Roth GA, Mensah GA, Abate YH, et al. Global burden of cardiovascular diseases and risks, 1990–2022. Journal of the American College of Cardiology. 2023; 82:2350–2473. DOI: 10.1016/j.jacc.2023.11.007 38092509 PMC7615984

[5] Mensah GA, Fuster V, Roth GA. A heart-healthy and stroke-free world. Journal of the American College of Cardiology. 2023; 82:2343–2349. DOI: 10.1016/j.jacc.2023.11.003 38092508

[6] UNCTAD, 2024.

[7] Andrade LD, Zanini V, Batilana AP, Carvalho ECAD, Pietrobon R, Nihei OK, et al. Regional disparities in mortality after ischemic heart disease in a Brazilian state from 2006 to 2010. PLoS ONE. 2013; 8:e59363. DOI: 10.1371/journal.pone.0059363 23527174 PMC3601963

[8] Kjærulff TM, Bihrmann K, Zhao J, Exeter D, Gislason G, Larsen ML, et al. Acute myocardial infarction: Does survival depend on geographical location and social background? Eur J Prev Cardiolog. 2019; 26:1828–1839. DOI: 10.1177/2047487319852680 31126196

[9] Dutra AC, Silva LL, Pedroso RB, Tchuisseu YP, Da Silva MT, Bergamini M, et al. The impact of socioeconomic factors, coverage and access to health on heart ischemic disease mortality in a Brazilian southern state: A geospatial analysis. Global Heart. 2021; 16:5. DOI: 10.5334/gh.770 33598385 PMC7824986

[10] Oyatani K, Koyama M, Himuro N, Miura T, Ohnishi H. Characterization of prehospital time delay in primary percutaneous coronary intervention for acute myocardial infarction: analysis of geographical infrastructure-dependent and -independent components. Int J Health Geogr. 2023; 22:7. DOI: 10.1186/s12942-023-00328-5 36998077 PMC10064653

[11] Chang J, Deng Q, Hu P, Guo M, Lu F, Su Y, et al. Geographic variation in mortality of acute myocardial infarction and association with health care accessibility in Beijing, 2007 to 2018. JAHA. 2023; 12:e029769. DOI: 10.1161/JAHA.123.029769 37301748 PMC10356049

[12] Oliveira M, Seringa J, Pinto FJ, Henriques R, Magalhães T. Machine learning prediction of mortality in Acute Myocardial Infarction. BMC Medical Informatics and Decision Making. 2023; 23:70. DOI: 10.1186/s12911-023-02168-6 37072766 PMC10111317

[13] Zhu X, Xie B, Chen Y, Zeng H, Hu J. Machine learning in the prediction of in-hospital mortality in patients with first acute myocardial infarction. Clinica Chimica Acta. 2024; 554:117776. DOI: 10.1016/j.cca.2024.117776 38216028

[14] Cano ALU, Jimena López-Mesa D, Elvira Alvarez-Rosero R, Alberto Garcés-Gómez Y. Predictive model for acute myocardial infarction in working-age population: A machine learning approach. International Journal of Electrical & Computer Engineering. 2024; 14(1):854–860. DOI: 10.11591/ijece.v14i1.pp854-860

[15] Barreto J, Silva JCQE, Sposito AC, Carvalho LS. O impacto da educação na mortalidade por todas as causas após infarto do miocárdio com supradesnivelamento do segmento ST (IAMCSST): Resultados do Brasília heart study. Arquivos Brasileiros de Cardiologia. 2021; 117:5–12. DOI: 10.36660/abc.20190854 34320060 PMC8294733

[16] Yufu K, Shimomura T, Kawano K, Sato H, Yonezu K, Saito S, et al. Usefulness of prehospital 12-Lead electrocardiography system in ST-Segment elevation myocardial infarction patients in Oita―Comparison between urban and rural areas, weekday daytime and weekday nighttime/holidays. Circ J. 2023; CJ-23–0365. DOI: 10.1253/circj.CJ-23-0365 37612071

[17] Duncan MS, Robbins NN, Wernke SA, Greevy RA, Jackson SL, Beatty AL, et al. Geographic ariation in access to cardiac rehabilitation. Journal of the American College of Cardiology. 2023; 81:1049–1060. DOI: 10.1016/j.jacc.2023.01.016 36922091 PMC10901160

[18] Bergamini M, Iora PH, Rocha TAH, Tchuisseu YP, Dutra ADC, Scheidt JFHC, et al. Mapping risk of ischemic heart disease using machine learning in a Brazilian state. PLoS ONE. 2020; 15:e0243558. DOI: 10.1371/journal.pone.0243558 33301451 PMC7728276

[19] Alanazi A, Nicholson N, Thomas S. The use of simulation training to improve knowledge, skills, and confidence among healthcare students: A systematic review. IJAHSP. 2017; 15(3). DOI: 10.46743/1540-580X/2017.1666

[20] Gianfrancesco MA, Tamang S, Yazdany J, Schmajuk G. Potential biases in machine learning algorithms using electronic health record data. JAMA Intern Med. 2018; 178:1544. DOI: 10.1001/jamainternmed.2018.3763 30128552 PMC6347576

[21] Bini SA. Artificial intelligence, machine learning, deep learning, and cognitive computing: What do these terms mean and how will they impact health care? The Journal of Arthroplasty. 2018; 33:2358–2361. DOI: 10.1016/j.arth.2018.02.067 29656964

[22] Boulos MNK, Peng G, VoPham T. An overview of GeoAI applications in health and healthcare. Int J Health Geogr. 2019; 18(7). DOI: 10.1186/s12942-019-0171-2 PMC6495523 31043176

[23] Collins GS, Reitsma JB, Altman DG, Moons K. Transparent reporting of a multivariable prediction model for individual prognosis or diagnosis (TRIPOD): The TRIPOD statement. BMC Med. 2015; 13:1. DOI: 10.1186/s12916-014-0241-z 25563062 PMC4284921

[24] Instituto Brasileiro de Geografia e Estatística (IBGE). De 2010 a 2022, população brasileira cresce 6.5% e chega a 203,1 milhões [Internet]. Gov.br; 2023a [cited 28 Aug 2023]. Available from: https://agenciadenoticias.ibge.gov.br/agencia-noticias/2012-agencia-de-noticias/noticias/37237-de-2010-a-2022-populacao-brasileira-cresce-6-5-e-chega-a-203-1-milhoes#:~:text=Em%201%C2%BA%20de%20agosto%20de,Brasil%20tinha%20203.062.512%20habitantes.

[25] Instituto Brasileiro de Geografia e Estatística (IBGE). Brasil em síntese: território – dados geográficos [Internet]. Gov.br; 2022a [cited 28 Aug 2023]. Available from: https://brasilemsintese.ibge.gov.br/territorio/dados-geograficos.html.

[26] Osho A, Fernandes MF, Poudel R, De Lemos J, Hong H, Zhao J, et al. Race-Based differences in ST-Segment–Elevation myocardial infarction process metrics and mortality from 2015 through 2021: An analysis of 178062 patients from the American Heart Association Get with the Guidelines–Coronary Artery Disease Registry. Circulation. 2023; 148:229–240. DOI: 10.1161/CIRCULATIONAHA.123.065512 37459415

[27] Brasil Ministério da Saúde. Departamento de Informação e Informática do Sistema Único de Saúde. Mortalidade- desde 1996 pela CID-10 [Internet]. Gov.br; 2023 [cited 28 Aug 2023]. Available: https://datasus.saude.gov.br/mortalidade-desde-1996-pela-cid-10.

[28] Luo W, Qi Y. An enhanced two-step floating catchment area (E2SFCA) method for measuring spatial accessibility to primary care physicians. Health & Place. 2009; 15:1100–1107. DOI: 10.1016/j.healthplace.2009.06.002 19576837

[29] Luo J, Chen G, Li C, Xia B, Sun X, Chen S. Use of an E2SFCA method to measure and analyse spatial accessibility to medical services for elderly people in Wuhan, China. IJERPH. 2018; 15:1503. DOI: 10.3390/ijerph15071503 30018190 PMC6068715

[30] Ibanez B, James S, Agewall S, Antunes MJ, Bucciarelli-Ducci C, Bueno H, et al. 2017 ESC guidelines for the management of acute myocardial infarction in patients presenting with ST-segment elevation. European Heart Journal. 2018; 39:119–177. DOI: 10.1093/eurheartj/ehx393 28886621

[31] Muñoz D, Roettig ML, Monk L, Al-Khalidi H, Jollis JG, Granger CB. Transport time and care processes for patients transferred with ST-Segment–Elevation myocardial infarction: the reperfusion in acute myocardial infarction in Carolina emergency rooms experience. Circ: Cardiovascular Interventions. 2012; 5:555–562. DOI: 10.1161/CIRCINTERVENTIONS.112.968461 22872054 PMC3600977

[32] Instituto Brasileiro de Geografia e Estatística (IBGE). População [Internet]. Gov.br; 2023b [cited 28 Aug 2023]. Available from: https://www.ibge.gov.br/geociencias/organizacao-do-territorio/malhas-territoriais.html.

[33] Brasil Ministério da Saúde. Departamento de Informação e Informática do Sistema Único de Saúde. Cadastro Nacional de Estabelecimentos de Saúde [Internet]. CNES; 2023c [cited 28 Aug 2023] Available from: https://cnes.datasus.gov.br/.

[34] Brasil Ministério da Saúde. Departamento de Informação e Informática do Sistema Único de Saúde. Morbidade Hospitalar do SUS (SIH/SUS) [Internet]. Gov.br; 2023d [cited 28 Aug 2023]. Available from: https://datasus.saude.gov.br/acesso-a-informacao/morbidade-hospitalar-do-sus-sih-sus/.

[35] Instituto Brasileiro de Geografia e Estatística (IBGE). Malhas territoriais [Internet]. Gov.br; 2023e [cited 28 Aug 2023]. Available from: https://www.ibge.gov.br/geociencias/organizacao-do-territorio/malhas-territoriais.html.

[36] Anselin L. GIS research infrastructure for spatial analysis of real estate markets. Journal of Housing Research. 1998; 9:113–133. https://www.jstor.org/stable/24833661.

[37] Moran PAP. Notes on continuous stochastic phenomena. Biometrika. 1950; 37:17–23. DOI: 10.2307/2332142 15420245

[38] Perobelli FS, Haddad EA. Padrões de comércio interestadual no Brasil, 1985 e 1997. Rev Econ Contemp. 2006; 10:61–88. DOI: 10.1590/S1415-98482006000100003

[39] Anselin L, Syabri I, Kho Y. GeoDa: An introduction to spatial data analysis. Geographical Analysis. 2006; 38:5–22. DOI: 10.1111/j.0016-7363.2005.00671.x

[40] Grekousis G, Feng Z, Marakakis I, Lu Y, Wang R. Ranking the importance of demographic, socioeconomic, and underlying health factors on US COVID-19 deaths: A geographical random forest approach. Health & Place. 2022; 74:102744. DOI: 10.1016/j.healthplace.2022.102744 35114614 PMC8801594

[41] Breiman L. Random forests. Machine Learning. 2001; 45:5–32. DOI: 10.1023/A:1010933404324

[42] Genuer R, Poggi JM. Random forests. In: Random Forests with R. Use R! Cham: Springer; 2020. pp. 33–55. DOI: 10.1007/978-3-030-56485-8_3

[43] Chan JYL, Leow SMH, Bea KT, Cheng WK, Phoong SW, Hong Z-W, et al. Mitigating the multicollinearity problem and its machine learning approach: A review. Mathematics. 2022; 10:1283. DOI: 10.3390/math10081283

[44] Lu B, Harris P, Charlton M, Brunsdon C. The GWmodel R package: Further topics for exploring spatial heterogeneity using geographically weighted models. Geo-spatial Information Science. 2014; 17:85–101. DOI: 10.1080/10095020.2014.917453

[45] Su Z, Lin L, Xu Z, Chen Y, Yang L, Hu H, et al. Modeling the effects of drivers on PM_2.5_ in the yangtze river delta with geographically weighted random forest. Remote Sensing. 2023; 15:3826. DOI: 10.3390/rs15153826

[46] Quiñones S, Goyal A, Ahmed ZU. Geographically weighted machine learning model for untangling spatial heterogeneity of type 2 diabetes mellitus (T2D) prevalence in the USA. Sci Rep. 2021; 11:6955. DOI: 10.1038/s41598-021-85381-5 33772039 PMC7997882

[47] Georganos S, Grippa T, Niang Gadiaga A, Linard C, Lennert M, Vanhuysse S, et al. Geographical random forests: a spatial extension of the random forest algorithm to address spatial heterogeneity in remote sensing and population modelling. Geocarto International. 2021; 36:121–136. DOI: 10.1080/10106049.2019.1595177

[48] Wang H, Seaborn T, Wang Z, Caudill CC, Link TE. Modeling tree canopy height using machine learning over mixed vegetation landscapes. International Journal of Applied Earth Observation and Geoinformation. 2021; 101:102353. DOI: 10.1016/j.jag.2021.102353

[49] LaZerte S. How to cite R and R packages. rOpenSci – open tools for open science; 2021 [cited 30 Jun 2024]. DOI: 10.59350/t79xt-tf203

[50] Belle V, Papantonis I. Principles and practice of explainable machine learning. Front Big Data. 2021; 4. DOI: 10.3389/fdata.2021.688969 PMC8281957 34278297

[51] Lotfata A, Moosazadeh M, Helbich M, Hoseini B. Socioeconomic and environmental determinants of asthma prevalence: A cross-sectional study at the U.S. county level using geographically weighted random forests. Int J Health Geogr. 2023; 22:18. DOI: 10.1186/s12942-023-00343-6 37563691 PMC10413687

[52] Yang Y, Sasaki K, Cheng L, Liu X. Gender differences in active travel among older adults: Non-linear built environment insights. Transportation Research Part D: Transport and Environment. 2022; 110:103405. DOI: 10.1016/j.trd.2022.103405

[53] Friedman JH. Greedy function approximation: A gradient boosting machine. Ann Statist. 2001; 29. DOI: 10.1214/aos/1013203451

[54] Georganos S, Stamatis K. A forest of forests: a spatially weighted and computationally efficient formulation of geographical random forests. ISPRS International Journal of Geo-Information. 2021; 11:471. DOI: 10.3390/ijgi11090471

[55] Garson GD. Data analytics for the social sciences: Applications in R. Abingdon, Oxon; New York, NY: Routledge, Taylor & Francis Group; 2022.

[56] Ali MM, Paul BK, Ahmed K, Bui FM, Quinn JMW, Moni MA. Heart disease prediction using supervised machine learning algorithms: Performance analysis and comparison. Computers in Biology and Medicine. 2021; 136:104672. DOI: 10.1016/j.compbiomed.2021.104672 34315030

[57] Krittanawong C, Virk HUH, Bangalore S, Wang Z, Johnson KW, Pinotti R, et al. Machine learning prediction in cardiovascular diseases: a meta-analysis. Sci Rep. 2020; 10:16057. DOI: 10.1038/s41598-020-72685-1 32994452 PMC7525515

[58] Cho SM, Austin PC, Ross HJ, Abdel-Qadir H, Chicco D, Tomlinson G, et al. Machine learning compared with conventional statistical models for predicting myocardial infarction readmission and mortality: A systematic review. Canadian Journal of Cardiology. 2021; 37:1207–1214. DOI: 10.1016/j.cjca.2021.02.020 33677098

[59] Larsen AI, Løland KH, Hovland S, Bleie Ø, Eek C, Fossum E, et al. Guideline-Recommended time less than 90 minutes from ecg to primary percutaneous coronary intervention for ST-Segment–Elevation myocardial infarction is associated with major survival benefits, especially in octogenarians: A contemporary report in 11226 patients from NORIC. JAHA. 2022; 11:e024849. DOI: 10.1161/JAHA.122.024849 36056722 PMC9496403

[60] Alizadeh R, Aghsaeifard Z, Sadeghi M, Hassani P, Saberian P. Effects of prehospital traige and diagnosis of ST segment elevation myocardial infarction on mortality rate. 2020; 13:569–575. DOI: 10.2147/IJGM.S260828 PMC7481285 32943908

[61] Meisel SR, Kleiner-Shochat M, Abu-Fanne R, Frimerman A, Danon A, Minha S, et al. Direct admission of patients with ST-Segment–Elevation myocardial infarction to the catheterization laboratory shortens pain-to-balloon and door-to-balloon time intervals but only the pain-to-balloon interval impacts short- and long-term mortality. JAHA. 2021; 10:e018343. DOI: 10.1161/JAHA.120.018343 33345559 PMC7955483

[62] Marrazzo G, Palermi S, Pastore F, Ragni M, Mauriello A, Zambrano A, et al. Enhancing ST-Elevation myocardial infarction diagnosis and management: The integral role of echocardiography in patients rushed to the cardiac catheterization laboratory. JCM. 2024; 13:1425. DOI: 10.3390/jcm13051425 38592271 PMC10931949

[63] Virani SS, Newby LK, Arnold SV, et al. 2023 AHA/ACC/ACCP/ASPC/NLA/PCNA guideline for the management of patients with chronic coronary disease: A report of the American Heart Association/American College of Cardiology Joint Committee on clinical practice guidelines. Circulation [internet]. Epub ahead of print. 2023 August 29; 148. DOI: 10.1161/CIR.0000000000001168 37471501

[64] Rafie N, Kashou AH, Noseworthy PA. ECG interpretation: Clinical relevance, challenges, and advances. Hearts. 2021; 2:505–513. DOI: 10.3390/hearts2040039

[65] de Oliveira GMM, Brant LCC, Polanczyk CA, et al. Estatística cardiovascular – Brasil 2020. Arq Bras Cardiol. 2020; 115:308–439. DOI: 10.36660/abc.20200812 33027364 PMC9363085

[66] Instituto Brasileiro de Geografia e Estatística (IBGE). Cor ou raça [Internet]. 2023 [cited 2023 Aug 28]. Available from: https://educa.ibge.gov.br/jovens/conheca-o-brasil/populacao/18319-cor-ou-raca.html.

[67] Instituto Brasileiro de Geografia e Estatística (IBGE). Pesquisa de pós-enumeração do censo demográfico 2022: Resultados da coleta e análise de conteúdo [Internet]. Rio de Janeiro: IBGE; 2024; [cited 2024 Jul 4]. Available from: https://www.ibge.gov.br/estatisticas/sociais/populacao/40418-pesquisa-de-pos-enumeracao-do-censo-demografico-2022.html.

[68] Verhagen MD, Brazel DM, Dowd JB, et al. Forecasting spatial, socioeconomic and demographic variation in COVID-19 health care demand in England and Wales [internet]. Epub ahead of print. 2020 March 21. DOI: 10.31219/osf.io/g8s96 PMC7321716 32594909

[69] Bennett M, Pistillo A, Recalde M, et al. Time trends in the incidence of cardiovascular disease, hypertension and diabetes by sex and socioeconomic status in Catalonia, Spain: A population-based cohort study. BMJ Open. 2023; 13:e066404. DOI: 10.1136/bmjopen-2022-066404 PMC10230898 37225269

[70] Shaik A, Chobufo MD, Gonuguntla K, Patel N, Sattar Y, Thyagaturu H, et al. Association between social vulnerability index and mortality following acute myocardial infarction in the US Counties. Current Problems in Cardiology. 2023; 48:101854. DOI: 10.1016/j.cpcardiol.2023.101854 37295635

